# Seasonal differences in the testicular transcriptome profile of free-living European beavers (*Castor fiber* L.) determined by the RNA-Seq method

**DOI:** 10.1371/journal.pone.0180323

**Published:** 2017-07-05

**Authors:** Iwona Bogacka, Łukasz Paukszto, Jan P. Jastrzębski, Joanna Czerwińska, Katarzyna Chojnowska, Barbara Kamińska, Aleksandra Kurzyńska, Nina Smolińska, Zygmunt Giżejewski, Tadeusz Kamiński

**Affiliations:** 1 Department of Animal Physiology, Faculty of Biology and Biotechnology, University of Warmia and Mazury in Olsztyn, Oczapowskiego 1A, Olsztyn, Poland; 2 Department of Plant Physiology and Biotechnology, Faculty of Biology and Biotechnology, University of Warmia and Mazury in Olsztyn, Oczapowskiego 1A, Olsztyn, Poland; 3 Institute of Animal Reproduction and Food Research of the Polish Academy of Sciences, Bydgoska 7, Olsztyn, Poland; Huazhong University of Science and Technology, CHINA

## Abstract

The European beaver (*Castor fiber* L.) is an important free-living rodent that inhabits Eurasian temperate forests. Beavers are often referred to as ecosystem engineers because they create or change existing habitats, enhance biodiversity and prepare the environment for diverse plant and animal species. Beavers are protected in most European Union countries, but their genomic background remains unknown. In this study, gene expression patterns in beaver testes and the variations in genetic expression in breeding and non-breeding seasons were determined by high-throughput transcriptome sequencing. Paired-end sequencing in the Illumina HiSeq 2000 sequencer produced a total of 373.06 million of high-quality reads. *De novo* assembly of contigs yielded 130,741 unigenes with an average length of 1,369.3 nt, N50 value of 1,734, and average GC content of 46.51%. A comprehensive analysis of the testicular transcriptome revealed more than 26,000 highly expressed unigenes which exhibited the highest homology with *Rattus norvegicus* and *Ictidomys tridecemlineatus* genomes. More than 8,000 highly expressed genes were found to be involved in fundamental biological processes, cellular components or molecular pathways. The study also revealed 42 genes whose regulation differed between breeding and non-breeding seasons. During the non-breeding period, the expression of 37 genes was up-regulated, and the expression of 5 genes was down-regulated relative to the breeding season. The identified genes encode molecules which are involved in signaling transduction, DNA repair, stress responses, inflammatory processes, metabolism and steroidogenesis. Our results pave the way for further research into season-dependent variations in beaver testes.

## Introduction

The European beaver (*Castor fiber* L.*)* is the largest rodent in Eurasia and the world’s second largest rodent after the capybara. In the past, the species was widely distributed across Eurasian forests, between western Europe and eastern Siberia, but it was nearly driven to extinction after centuries of hunting for its fur and castoreum, the exudate from the beaver scent gland. By the end of the 19^th^ century, hunting pressure combined with the decline in beaver habitats reduced the Eurasian beaver population to 1,200 individuals in several remote regions [1]. Fortunately, the introduction of a hunting ban and other conservation measures enabled the beaver population to survive and spread to other regions through natural and artificial recolonization.

The beaver population in Europe has increased significantly in the last two decades, and the conservation status of the species on the Red List of Threatened Species of the International Union for Conservation of Nature (IUCN) has been changed from endangered to least concern. The beaver is often referred to as an ecosystem engineer in Eurasian temperate forests [2, 3, 4] due to its ability to create or modify habitats. Beavers enhance biodiversity and prepare the ecosystem for the emergence of other species of plants, water-dwelling invertebrates, fish, amphibians, reptiles, birds and mammals [3–6]. Beaver dams retain sediments and organic material [3], they influence the nutrient cycle and speed up the decomposition of organic matter. The dams also induce changes in the chemical and physical parameters of water [3, 7].

Beavers are monogamous animals, and both males and females co-parent their young. Beavers usually live in small family groups of 2–14 individuals which consist of an adult pair, offspring younger than one year, yearlings and, sporadically, subadults [8]. They have a seasonal pattern of reproduction. Similarly to hamsters and horses, beavers are long-day breeders, and their cyclicity is dependent on photoperiod. The reproductive activity of beavers peaks in winter, and the animals mate under the ice between January and March. In summer, beavers take care of their offspring and store food reserves. Beavers do not hibernate, and they accumulate subcutaneous fat depots in autumn [1, 9]. Both males and females exhibit reproductive seasonality, but males appear to have a longer breeding season because the completion of spermatogenesis usually requires more time than oogenesis [10] and earlier recrudescence of male breeding activity facilitates sexual competition for mates [11]. Testis size, the ability to produce gametes, hormonal activity and gene expression change throughout the year in seasonally active males, especially in wild animals [12–15].

In view of the above findings, we hypothesized that changes in gene expression between breeding and non-breeding seasons can also be detected in beaver testes. In the presented study, beaver testes harvested in two periods of reproductive activity were subjected to comprehensive transcriptomic analyses with the use of the RNA-Seq approach. A differential expression analysis of testes harvested in breeding (April) and non-breeding (November) seasons was carried out to determine the impact of reproductive activity on transcriptome profiles. Differentially expressed genes and their products were linked with biological functions.

## Materials and methods

### Animals

The study was performed on European beavers captured in various habitats in the Region of Warmia and Mazury in north-eastern Poland, as described in our previous studies [16, 17]. The animals were captured upon the prior consent of the Regional Directorate for Environmental Protection in Olsztyn, Poland (decision No. RDOS-28-OOP-6631-0007-638/09/10/pj). All procedures were conducted in accordance with the ethical standards of the Institutional Animal Ethics Committees (local approvals: SGGW/11/2010 and UWM/87/2012/DTN). Beavers were captured by the same group of qualified staff over a period of two years (2011–2012), in the same period of the year, during two different stages of reproductive activity: in April–‘breeding period’ (n = 4) and November–‘non-breeding’, sexual silence (n = 4). Every experimental group consisted of different individuals. The animals were placed in cages and transported to the laboratory of the Research Station for Ecological Agriculture and Preservation of Native Breeds of the Polish Academy of Sciences in Popielno. Each beaver was weighed in the cage, and the weight of an empty cage was subtracted. Beavers were anesthetized by injection of xylazine (3 mg/kg BW; Sedazin®, Biovet Puławy, Poland) and ketamine (15 mg/kg BW; Bioketan, Vetoquinol Biowet, Poland) and sacrificed by exsanguination. The testes were sampled, frozen immediately in liquid nitrogen and stored at -80°C until total RNA isolation.

### RNA isolation and transcriptome sequencing

Total RNA for transcriptome sequencing was isolated from four testicular compartments (n = 2 for each experimental group) using the Qiagen RNeasy Kit and the Qiagen RNase-Free DNase Set for cleanup (Qiagen, USA) according to the manufacturer’s recommendations. The concentration and purity of total RNA isolated from testicular tissues were analyzed with the Tecan Infinite M200 plate reader (Tecan Group Ltd, Switzerland), and RIN values were evaluated in the Agilent Bioanalyzer 2100 (Agilent Technology, USA). Total RNA was isolated using an OpenExome kit (Poland) to prepare sequencing libraries. The cDNA library was developed based on total RNA with the TruSeq Stranded mRNA LT Sample Prep Kit v3, using the appropriate index adapters for each sample. The concentration of mRNA ranged from 305 to 348 ng/μl. Libraries were pooled and sequenced to produce 2 x 100 bp paired-end (PE) reads in the Illumina HiSeq 2000 high-throughput sequencer. All samples were sequenced in a single lane with human reference mRNA as positive control. Libraries were quantified with the use of the KAPA library quantification kit.

### *De novo* transcriptome assembly

The quality of raw paired-end reads was evaluated with the use of the FastQC software v.0.10.0 (www.bioinformatics.babraham.ac.uk). Adaptor sequences were removed from raw sequencing reads with the AlienTrimmer tool in default mode [18]. Low-quality reads (PHRED <20) were removed from the dataset. Trimmed sequences from 4 testis samples were *de novo* assembled in the Trinity software v. r20140717 [19] with a 20-core processor and a 90 GB RAM server of the Regional IT Center (Olsztyn, Poland). The reconstructed contigs were used to develop a reference transcriptome of *Castor fiber* with a minimum transcript length of 500 nt. *De novo* assembly was performed with the Trinitystats.pl script, and the assembly statistics included average contig length, GC content, total assembled bases and N50 parameters. Duplicate transcripts were removed from the reference transcriptome in the CD-HIT-EST v.4.6 software (http://weizhong-lab.ucsd.edu/cd-hit/) to minimize data redundancy (by clustering sequences at 95% identity) [20]. The Benchmarking Universal Single-Copy Orthologs (BUSCO) v.1.1 tool [21] was used to check the completion of transcriptome assembly based on the percentage of transcripts identified as putative core eukaryotic genes (CEGs).

### Comprehensive transcriptome analysis

The assembled transcripts with FPKM > 2 in at least two probes were used in a comprehensive analysis because this approach proved to be highly effective in other studies [22, 23]. Selected contigs were blasted (blastx, cut-off E value of 1e-5) against five ENSEMBL databases of protein sequences from *Mus musculus*, *Rattus norvegicus*, *Homo sapiens*, *Ictidomys tridecemlineatus* and *Dipodomys ordii*. The highly expressed transcripts (FPKM >2) were aligned with the NCBI database of rodent proteins with the use of the CloudBlast (blastx-fast) tool implemented in BLAST2GO to obtain genes specific for beaver testes [24]. Transcripts were annotated to three main gene ontology categories: biological process (BP), cellular component (CC) and molecular function (MF). The search for conserved protein motifs and the prediction of protein families were performed in the InterProScan v.5 program [25] for each highly expressed transcript.

The obtained beaver contigs were compared with the rat genome to determine the abundance and distribution of transcripts. Contigs with FPKM >2 and longer than 500 bp were used as search queries in blastx against the NCBI nr protein database to identify testicular unigenes. Unigenes were annotated with the BLAST tool against the nr database with cut-off E-value of 10^−5^. The obtained unigenes were mapped onto the *Rattus norvegicus* genome, a model organism that is most closely related to the beaver, with the use of the GMAP software [26]. Each unigene was compared with the rat genome. Based on this alignment, the reciprocal best-hits method with a 100 kb window was used to determine the distribution of beaver transcripts across the rat genome. The RBH graph was generated in the Circos program [27].

### Analysis of gene expression

Trimmed paired-end reads of each sample were realigned to a non-redundant beaver transcriptome in the Bowtie software [28]. Mapped contigs were filtered to remove transfrags with low expression levels. Non-normalized read counts and TMM normalized FPKM were calculated in the eXpress software [29] to estimate transcript expression levels.

The expression of contigs with absolute log fold-change (logFC) of 2 or higher and the corrected p-value (FDR) of less than 0.05 was significantly different. Two statistical methods were used to confirm differentially expressed transcripts in the testes (autumn vs. spring): DESeq [30] and edgeR [31]. Differentially expressed contigs were aligned to the NCBI nr protein database with blastx (cut-off E-value of 10^−5^) to identify the coding DNA sequence (CDS). Differentially expressed genes (DEGs) were visualized in a heatmap plot with gplots in R package (http://www.r-project.org/). All DEGs were compared against the *Homo sapiens* database in the KEGG Orthology Based Annotation System (KOBAS) for metabolic pathway and Gene Ontology enrichment analyses with p-value < 0.05 and q-value < 0.9.

### RNA isolation, cDNA synthesis and qRT-PCR for validation of transcriptome sequencing data

Total RNA was isolated from beaver testes collected during breeding (n = 4) and non-breeding seasons (n = 4) using the A&A mini column kit (A&A Biotechnology, Poland) with a DNase treatment step. The concentration and the amount of the isolated RNA were determined spectrophotometrically (Infinite M200 PRO, Tecan, Switzerland), and RNA integrity was verified on 1.5% agarose gel. One μg of RNA was reverse transcribed into cDNA with a total volume of 20 μL with the use of the QuantiTect® Reverse Transcription Kit (Qiagen, USA). The reaction included two steps: preliminary genomic DNA elimination (2 min at 42°C) and main reverse transcription (30 min at 42°C). The reaction was terminated by incubating the samples for 3 min at 95°C in a thermal cycler (Eppendorf, Germany).

To validate transcriptome sequencing results, the expression of differentially regulated genes, such as *PRRKG1*, *DSG2*, *SMARCA2*, *BMX* and *AGT*, was determined by qRT-PCR with the use of the 7300 PCR System and the Power SYBR Green Master Mix (Applied Biosystems, USA). The constitutively expressed *ACTB* and *GAPDH* genes were used as reference (housekeeping) genes according to a previously described procedure [32, 33]. Specific primer sequences (S1 Table) for amplifying target and reference genes were designed in the Primer Express Software 3 (Applied Biosystems). PCR reaction mixtures with a final volume of 25 μL consisted of cDNA (20 ng for target genes and 5 ng for reference genes), 400 nM of the primers, 12.5 μL of the Power SYBR Green PCR Master Mix (Applied Biosystems, USA), and RNase-free water. The qRT-PCR reaction conditions were as follows: enzyme activation and initial denaturation at 95°C for 10 min, followed by 40 cycles of denaturation at 95°C for 15 s, annealing for 1 min at 60°C for all tested genes. In no template controls (NTC), cDNA was replaced with RNase-free water. The efficiency of qRT-PCR for each tested cDNA was determined at 100%. All samples were run in duplicates. The specificity of amplification was tested at the end of the reaction by analyzing the melting-curve. The relative expression of the tested genes was calculated with the use of the comparative cycle threshold method (ΔΔCt) described by Schmittgen & Livak [34], where ΔΔCt is obtained by subtracting ΔCt of the geometric mean, calculated from the Ct value of both reference genes (*GAPDH* and *ACTB*), from the corresponding ΔCt of each experimental sample of the target gene. The group with the lowest expression was selected as the calibrator. The results of qRT-PCR were processed statistically in the Statistica software (Statoft Inc. Tulsa, USA) with the use of the Student’s t-test and were expressed as means ± SEM. The results were regarded as statistically significant at p < 0.05.

## Results

### *De novo* transcriptome assembly

A total of 380.22 (2 x 190.11) million of paired-end reads were sequenced in the Illumina HiSeq 2000 sequencer for the *de novo* assembly of the transcriptome of *Castor fiber* testicular tissue (Table 1).

**Table 1:** Sequencing and quality control results.

Sample name	Number of raw reads	GC content [%]	trimmed reads	percentage of bases with quality score >20 [%]	percentage of bases with quality score >30 [%]	QMAP >30 (percentage of mapped reads) [%]
**Cf_s1**	86,987,736	47.1	85,385,030	97.5	94.9	89.7
**Cf_s2**	117,250,752	51.8	115,760,900	97.9	95.7	91.5
**Cf_a1**	81,113,500	47.3	79,295,976	97.2	94.5	88.7
**Cf_a2**	94,863,184	46.7	92,621,004	97.1	94.1	89.2

Sample names: Cf_s1, Cf_s2 –*Castor fiber* during spring (samples 1 and 2); Cf_a1, Cf_a2 –*Castor fiber* during autumn (samples 1 and 2).

After processing of raw reads (adaptor sequences were cut and low-quality reads with Phred value < 20 were removed), 373.06 million (98.12%) clean reads were obtained and assembled *de novo* using the Trinity software. Assembly results are summarized in Table 2. The assembly was performed on 260,311 contigs. To minimize redundancy, contigs with sequence similarity higher than 95% were clustered in 231,045 non-redundant contigs grouped into 130,741 unigenes. Each unigene was described by a group of related transcripts included in the same de Bruijn graph, and each cluster was regarded as a single gene. Non-redundant contigs ranged from 500 nt to 20,720 nt, with N50 value (the length of the contig describing half of the sum of the lengths of all contigs) of 1,734 nt. The longest transcript was identified as fibrous sheath interacting protein 2 with 99% coverage and 69% sequence identity with *Marmota marmota* (using blastx, NR Rodentia). More than 100 k contigs were longer than 1000 nt. A total of 39,657 unigenes with ORFs longer than 300 nt were detected.

**Table 2:** Statistics of transcriptome assembly.

Number of trimmed reads used for the assembly	373,062,910
Number of contigs	260,311
Number of non-redundant contigs (sequence with >95% identity were filtered out)	231,045
Number of “unigenes” (isoform clusters)	130,741
Percentage of GC	46.51
Total size of contigs (Mb)	316.37
Total size of the longest isoforms per “unigene” (Mb)	163.40
Average contig length (nt)	1,369.3
Median contig length (nt)	931
N50 (nt)	1,734
Longest contig	20,720
Number of contigs > 1K nt	105,848
Number of contigs > 10K nt	213

### Comprehensive analysis of beaver testicular transcriptome

In the set of all non-redundant contigs, 88% (2663/3023) complete and 4.4% (136/3023) fragmented core eukaryotic (vertebrate) genes were found. More than 92% of the predicted housekeeping genes confirmed the completeness of transcriptome assembly.

**Fig 1:**
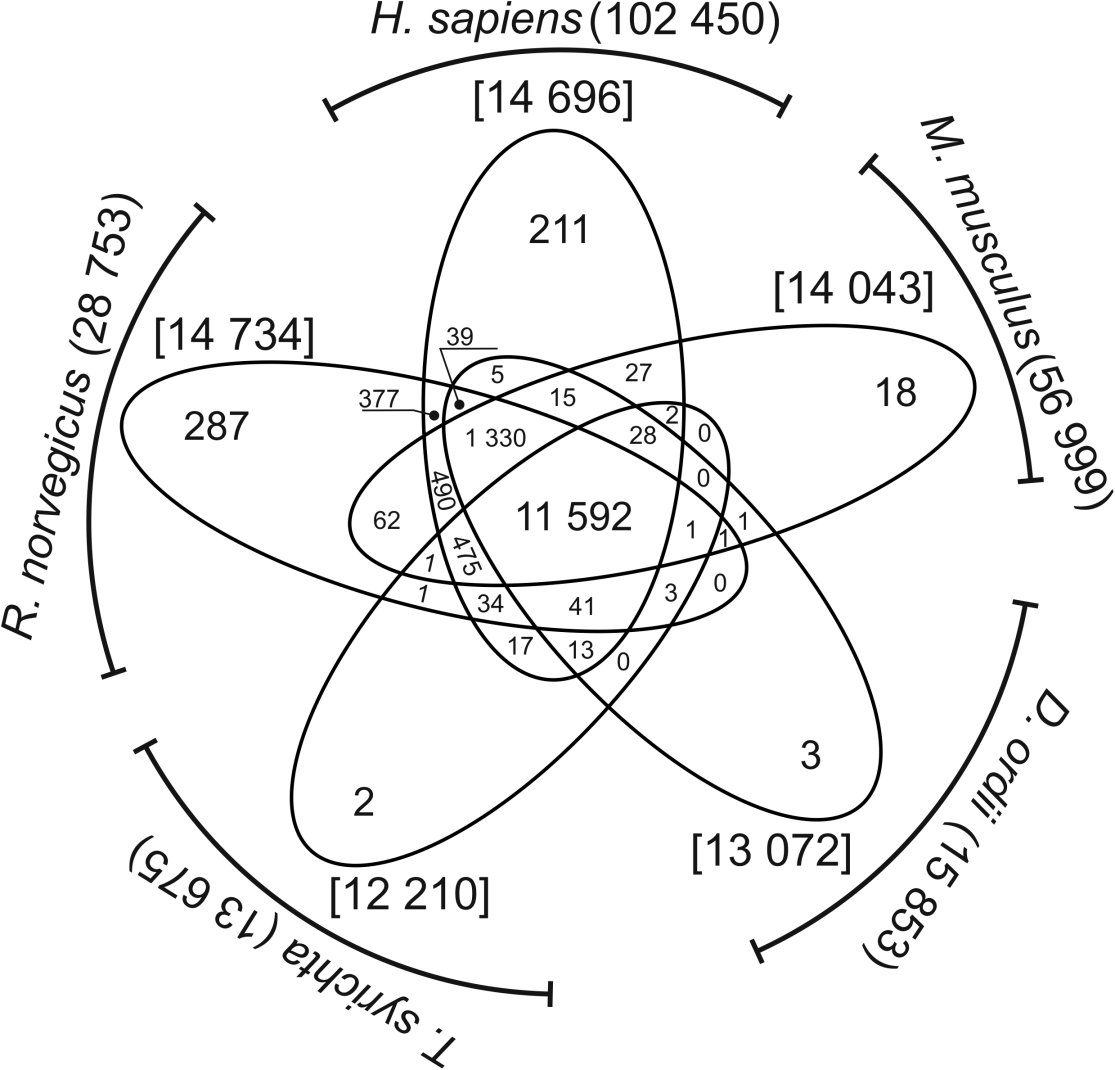
Venn diagram—Summary of annotations of highly expressed testicular transcripts (FPKM >2) in *Castor fiber*. Based on sequence homology, *Castor fibe*r transcripts (FPKM >2) were matched to 5 species (4 Rodentia species and *Homo sapiens*). The numbers in the overlapping areas denote BLASTX matches with two or more species. The numbers in the non-overlapping areas denote uniquely blasted homologs.

The whole set of clean reads was aligned back (with MAPQ >30) to the non-redundant *Castor fiber* transcriptome, and 88.7% (Cf_a1) to 91.5% (Cf_s2) of reads were successfully mapped (Table 2). These data were used for transcriptome profiling (comprehensive analysis) and differential expression analyses. Gene counts and TMM normalized FPKM values were calculated with the eXpress tool, and transcript expression levels were estimated. To remove weakly expressed transcripts from assembly datasets, only contigs with FPKM > 2 in at least 50% of the samples were used in comprehensive analysis. In the group of 26,228 unigenes that fulfilled the above requirement, the longest isoforms were extracted and blasted (cut-off E-value of 10e-5) with five protein Ensembl databases. The blastx search revealed 14,734, 14,696, 14,043, 13,072 and 14,065 highly expressed homologs for *Rattus norvegicus*, *Ictidomys tridecemlineatus*, *Mus musculus*, *Dipodomys ordii* and *Homo sapiens*, respectively. In this group, 258 corresponding homologs were identified exclusively in rat, 33 in squirrel, 16 in mouse, and 1 in kangaroo rat. The remaining 172 beaver transcripts were not found in Rodentia, and they had only human homologs (Fig 1).

**Fig 2:**
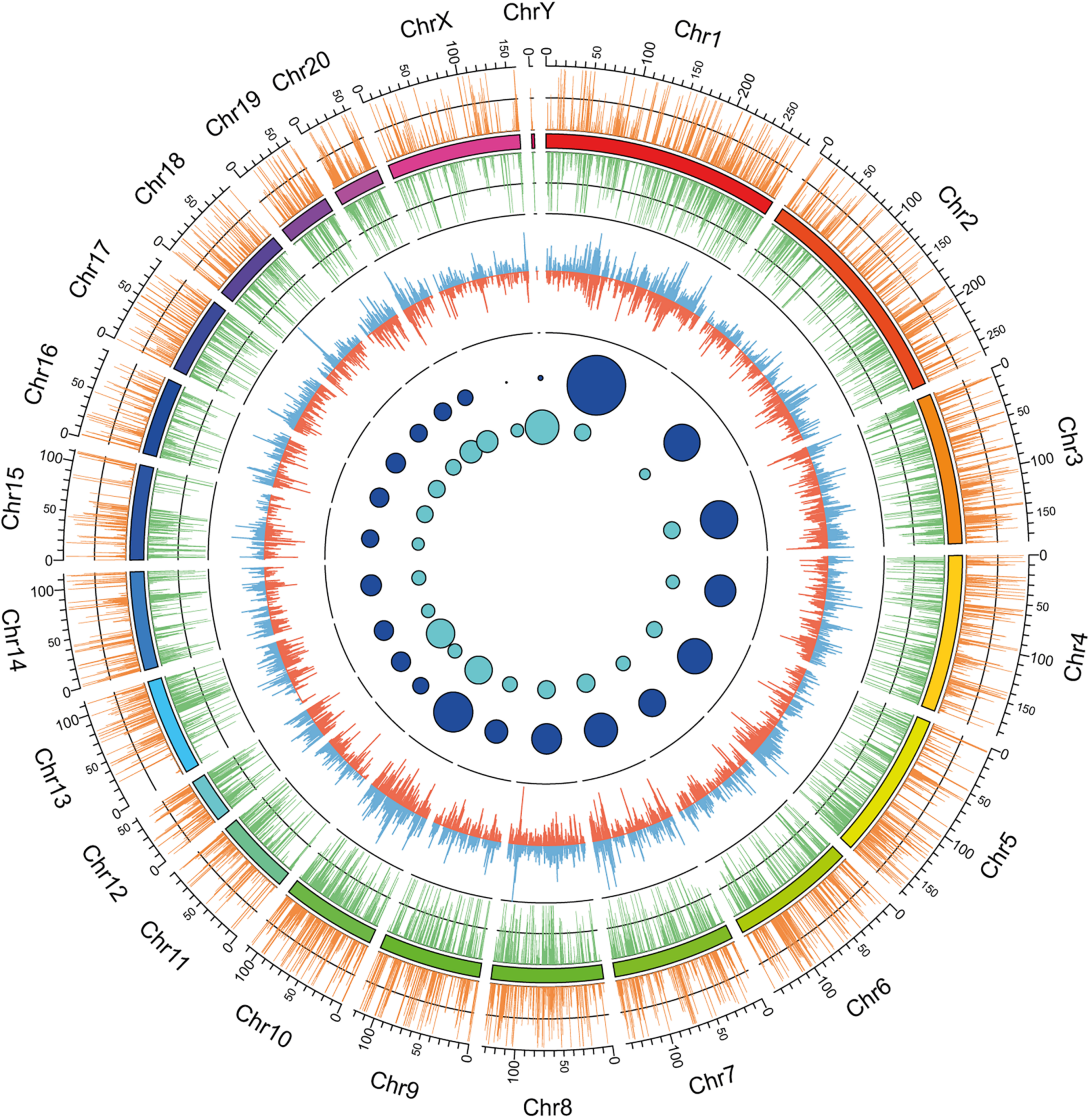
Abundance and distribution of Castor fiber unigenes compared with the Rattus norvegicus genome. The outer track shows the density of mapped unigenes of C. fiber to the R. norvegicus genome, in both forward (orange histogram) and reverse (green histogram) strands. The middle track represents exome density throughout R. norvegicus chromosomes in both forward (blue histogram) and reverse (red histogram) strands. The dark blue circles in the inner track depict the percentage of all unigenes aligned to each chromosome (the size of circle corresponds to the percentage of aligned unigenes), whereas the light blue circles depict the density of identified unigenes throughout chromosomes (the size of circle corresponds to the amount of aligned unigenes per chromosome length). The chromosomes length is expressed in million base pairs (Mb).

Highly expressed transcripts were compared with the rat genome (the best described Rodentia genome with the closest phylogenetic relationship with *Castor fiber*). A total of 15,774 unigenes were annotated using the NCBI nr database. The results of contig mapping to the rat genome are visualized in Fig 2. Approximately 60.14% of highly expressed transcripts longer than 500 bp with FPKM>2 were mapped to orthologs localized on the rat genome. A comprehensive analysis revealed that transcriptome assembly had a broad representation and could be used as the reference transcriptome for *Castor fiber* testicular tissue.

### Functional classification

**Fig 3:**
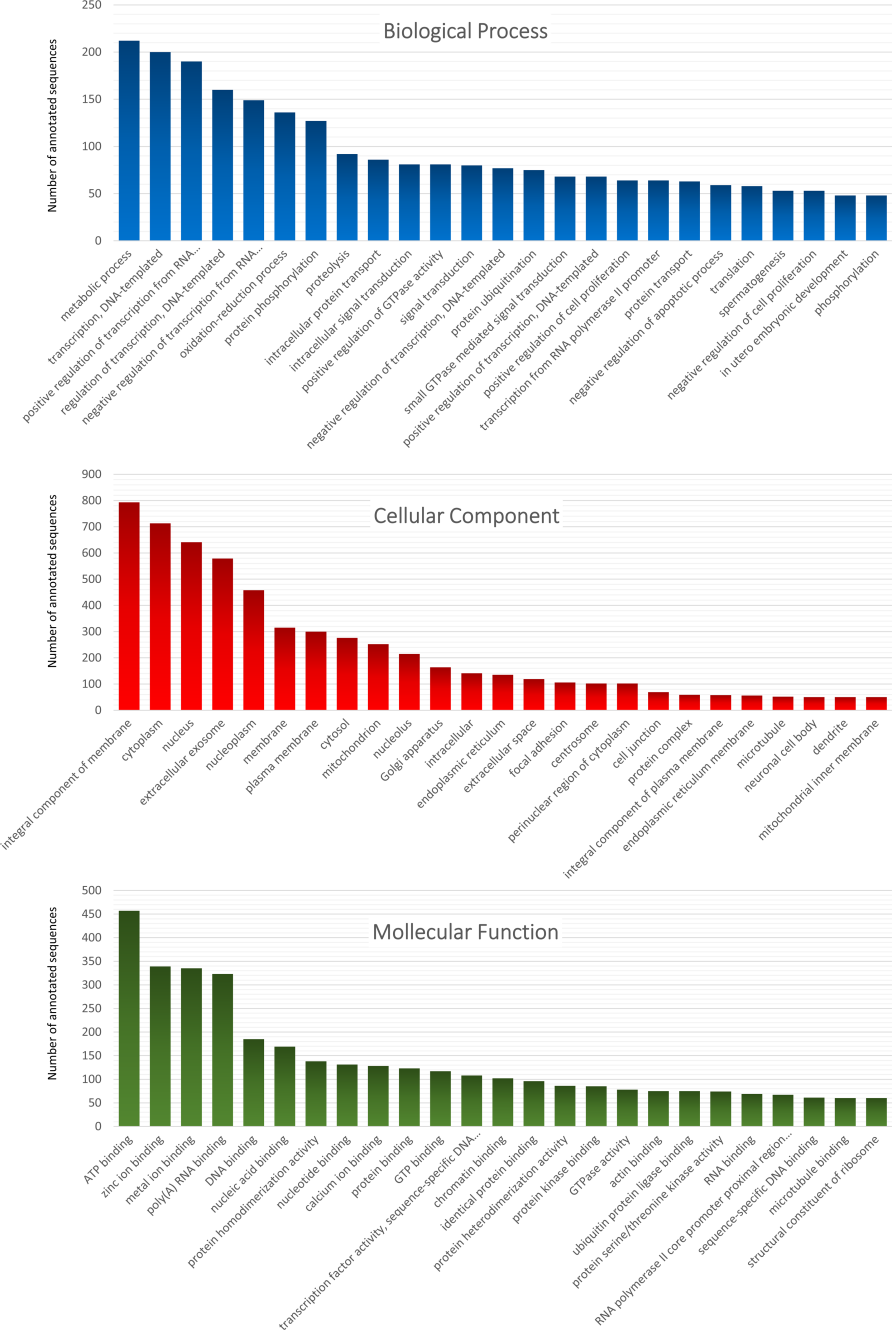
Top 25 gene ontology annotations for the assembled transcriptome. Annotations (received by Blast2GO tool from ‘no.’ NCBI database limited to [rodentia, taxa:9989] using blastx-fast algorithm) are divided into three GO categories: Biological Process (the upper part, in blue), Cellular Component (the middle part, in red), Molecular Function (the lower part, in green). Values on the vertical axis indicate the number of sequences assigned to GO terms. Top 25 GO terms are presented on the vertical axis.

The study revealed 8,188 highly expressed genes mapped to GO terms, where 3918 were assigned to the Biological Process sub-ontology (Fig 3). The annotated pathways included metabolic processes represented by 212 unigenes (5.41%), transcription, DNA-templated–represented by 200 unigenes (5.10%), regulation of transcription, DNA-templated–represented by 160 unigenes (4.08%), and positive and negative regulation of transcription from RNA polymerase–represented by 190 and 149 unigenes, respectively (4.85% and 3.80%). The most interesting top-hit biological process was spermatogenesis, represented by 53 corresponding unigenes (S2 Table). Other biological processes related to the male gonadal tissue included 23 (spermatoid development), 17 (steroid hormone mediated signaling pathway), 15 (sperm motility), 13 (male gonad development), 5 (Sertoli cell development), 3 (Sertoli cell proliferation), 3 (sperm axoneme assembly) and 2 (spermatid differentiation) highly expressed unigenes (S2 Table).

In the cellular component category, the highest number of transcripts matched the term integral component of membrane (793), cytoplasm (713), nucleus (641) and extracellular exosome (579). The molecular function category consisted of 1885 terms which were assigned to 3819 unigenes. In this category, the majority of unigenes (457) were associated with ATP binding (Fig 3).

A total of 423 unigenes were classified into 116 KEGG pathways (S3 Table). The top three KEGG pathways involved purine metabolism (312, 73.75%), thiamine metabolism (245, 57.91%) and aminobenzoate degradation (73, 17.26%) (S1 Fig). A metabolic pathway analysis revealed 12 highly expressed transcripts that were involved in steroid hormone biosynthesis, 3 transcripts that were involved in steroid biosynthesis and 2 transcripts that were involved in steroid degradation (S4 Table).

The majority of functional annotation results (3,699 out of 8,188) matched *Ictidomys tridecemlineatus*. InterPro domain annotations showed functional information for 13,069 unigenes. Most of the unigenes annotated in the InterProScan were found in protein domain and functional databases: 10,578 PFAM, 3249 PANTHER and 5279 SMART. The identified proteins (encoded by unigenes) also encompassed 115 oxidoreductases, 403 transferases, 326 hydrolases, 30 lyases, 35 isomerases and 54 ligases.

### Seasonal differences in beaver testicular transcriptome

**Fig 4:**
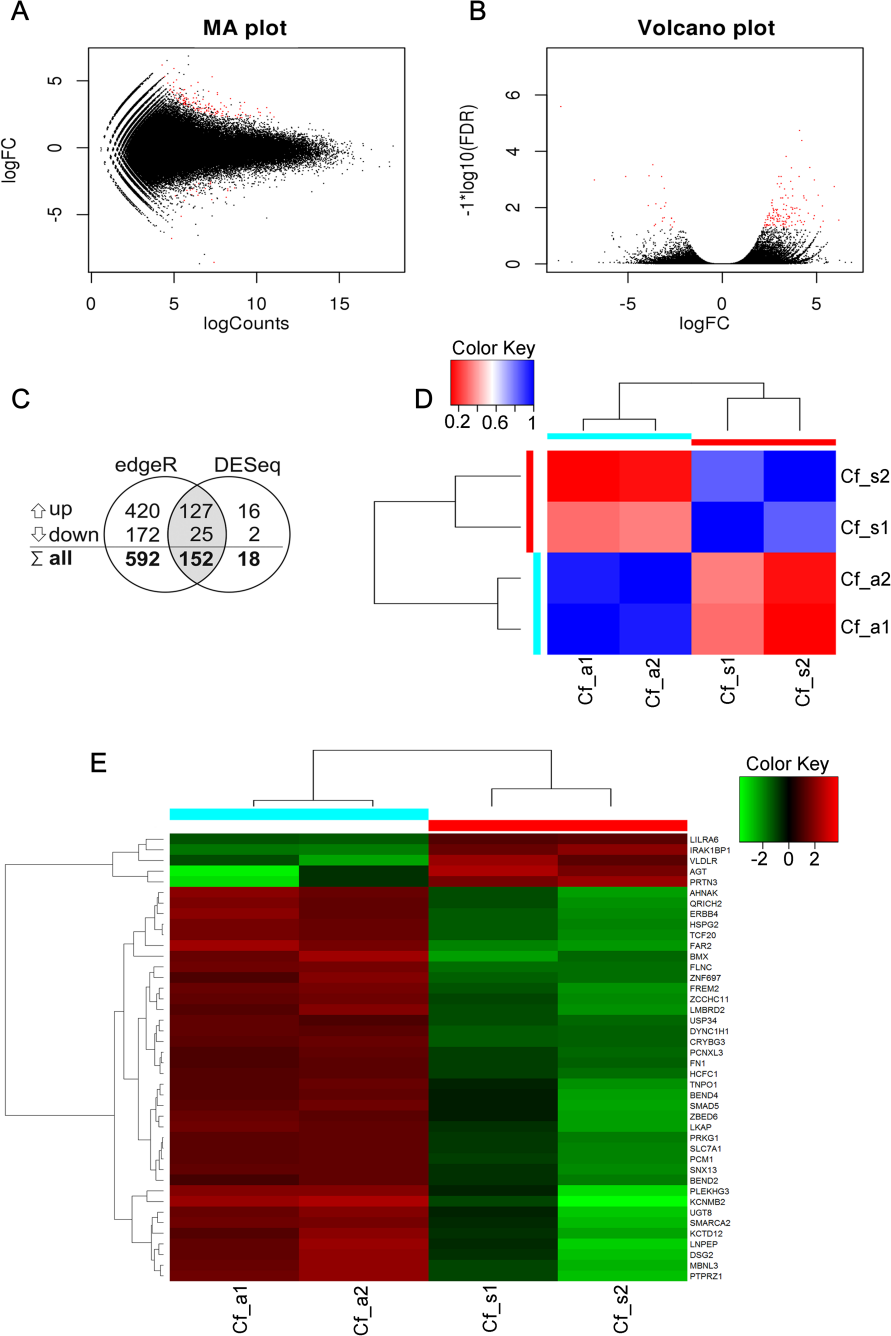
Analyses of different gene expression levels in breeding and non-breeding seasons. MA plot (A) and volcano plot (B) describe log2 fold change between spring and autumn according to DESeq. Each point represents one contig. The red points represent the contigs of p adjusted < 0.05 and log2 fold change > 1. (C)–represents the Venn diagram of the quantity of DEGs calculated by both methods: edgeR and DESeq (log2 fold change > 1, p adjusted < 0.05). Values in the "up" line denote the number of up-regulated genes, values in the "down" line denote the number of down-regulated genes, and values in the "all" line denote the total number of down- and up-regulated genes. (D)–represents the matrix of Pearson correlation coefficients calculated for 152 contigs designated as significantly expressed using the DESeq statistical method. Blue color represents high correlation and red–low samples correlation. Samples are clustered by both rows and columns. Cyan and red bars indicate the samples grouped by seasons. E–represents the heat map of differences in the expression of 42 genes between spring and autumn. The rows represent selected genes; in each column, individual samples are grouped into two seasons (upper dendrogram): spring (red bar) and autumn (cyan bar). The colors and intensity of fields in the heat map indicate transformed fold change (log2 fold change values from -3,767 to 3,767): red—underexpression, green—overexpression. The gene order was generated automatically in the dendrogram (left side of the graph).

A comparison of the testes harvested during breeding (April) and non-breeding (November) seasons revealed 152 transcripts (including 42 protein coding genes) that were significantly differentially expressed (log2FC >1; FDR 0.05) (Fig 4). The remaining non-annotated differentially expressed contigs were putative noncoding RNAs or novel genes. Of these, 37 genes were up-regulated and 5 genes were down-regulated in beaver testes from the non-breeding season relative to the samples obtained in the breeding season (Table 3).

**Table 3:** Seasonal changes in gene expression in beaver testes.

Gene symbol	id	FC	log2FC	pval	padj	Cf_s1	Cf_s2	Cf_a1	Cf_a2
**FN1**	c90752_g1	4.776	2.256	5.06E-05	4.21E-02	2.230	1.360	7.045	5.937
**BEND2**	c63053_g2	4.783	2.258	4.90E-05	4.10E-02	2.045	0.664	6.634	7.452
**BEND4**	c85676_g2	4.926	2.300	2.33E-05	2.70E-02	6.742	1.318	20.886	20.474
**PCNXL3**	c91386_g2	5.017	2.327	4.57E-05	4.01E-02	1.573	0.791	6.529	6.757
**HCFC1**	c83255_g1	5.237	2.389	4.88E-05	4.10E-02	1.007	0.359	3.613	3.417
**TNPO1**	c91956_g1	5.258	2.395	1.29E-05	2.01E-02	14.297	3.385	48.846	49.743
**ZBED6**	c91632_g3	5.300	2.406	1.60E-05	2.25E-02	5.406	0.791	17.560	13.601
**PRKG1**	c90317_g3	5.578	2.480	1.15E-05	1.92E-02	2.333	0.833	10.046	9.306
**USP34**	c94489_g13	5.622	2.491	1.04E-05	1.80E-02	2.086	1.202	12.484	8.688
**SMAD5**	c91171_g5	5.628	2.493	8.58E-06	1.58E-02	2.364	0.443	7.819	8.504
**SLC7A1**	c89116_g2	5.662	2.501	4.79E-05	4.10E-02	0.524	0.179	2.265	2.172
**SNX13**	c91327_g1	5.912	2.564	1.56E-05	2.24E-02	2.333	0.685	10.352	8.997
**PCM1**	c90381_g1	6.108	2.611	1.97E-06	6.71E-03	7.770	2.773	37.251	33.747
**DYNC1H1**	c92105_g6	6.198	2.632	2.93E-06	8.51E-03	0.843	0.569	5.735	4.421
**CRYBG3**	c82417_g2	6.288	2.653	1.47E-06	5.33E-03	1.562	1.076	9.769	9.537
**LILRA6**	c94606_g8	0.155	-2.685	1.85E-05	2.43E-02	3.546	2.657	0.593	0.531
**LKAP**	c116021_g1	6.537	2.709	5.73E-06	1.18E-02	5.622	1.244	26.784	20.735
**KCTD12**	c73375_g1	7.378	2.883	6.27E-06	1.26E-02	2.189	0.538	7.475	11.198
**ZCCHC11**	c88818_g14	7.457	2.899	2.01E-05	2.48E-02	1.398	0.506	7.877	7.636
**SMARCA2**	c80237_g2	7.529	2.913	2.26E-07	1.41E-03	13.464	2.415	64.991	60.178
**UGT8**	c80701_g1	7.557	2.918	2.78E-05	3.03E-02	0.421	0.053	1.692	1.902
**QRICH2**	c70131_g1	8.024	3.004	8.44E-08	7.88E-04	5.735	2.003	41.438	26.865
**LMBRD2**	c91156_g7	8.200	3.036	3.21E-07	1.83E-03	0.545	0.200	2.829	3.871
**ZNF697**	c80606_g1	8.202	3.036	2.03E-05	2.48E-02	0.606	0.380	3.785	5.666
**DSG2**	c14706_g1	8.701	3.121	6.82E-08	7.88E-04	1.069	0.190	4.779	6.593
**FREM2**	c92500_g5	8.724	3.125	2.12E-07	1.39E-03	0.206	0.084	1.415	1.371
**FLNC**	c85935_g2	9.121	3.189	1.23E-06	4.58E-03	0.154	0.116	1.501	1.380
**LNPEP(IRAP)**	c81892_g2	9.156	3.195	1.09E-06	4.52E-03	0.216	0.032	0.937	1.371
**TCF20**	c81986_g1	9.331	3.222	4.44E-05	3.98E-02	0.452	0.190	3.986	3.002
**PLEKHG3**	c86615_g2	9.404	3.233	6.13E-05	4.82E-02	0.514	0.032	2.724	2.462
**HSPG2**	c87960_g1	9.419	3.236	4.00E-05	3.73E-02	0.062	0.032	0.535	0.425
**ERBB4**	c83215_g2	10.308	3.366	2.93E-05	3.05E-02	0.134	0.063	1.424	0.782
**MBNL3**	c91522_g7	10.407	3.380	5.86E-09	1.53E-04	1.716	0.411	9.960	13.524
**AHNAK**	c90346_g2	10.595	3.405	9.65E-06	1.70E-02	0.421	0.169	4.034	2.220
**VLDLR**	c87675_g1	0.085	-3.549	4.27E-05	3.89E-02	6.372	2.425	0.612	0.203
**BMX**	c77554_g2	12.756	3.673	2.35E-08	3.84E-04	0.370	0.527	5.506	7.964
**IRAK1BP1**	c90008_g2	0.078	-3.689	1.37E-08	2.97E-04	16.064	17.746	2.179	1.602
**PTPRZ1**	c73074_g1	14.635	3.871	4.48E-05	3.98E-02	0.062	0.011	0.421	0.521
**AGT**	c79569_g2	0.068	-3.881	5.13E-08	7.44E-04	7.534	3.290	0.105	0.705
**PRTN3**	c51671_g1	0.066	-3.931	3.00E-06	8.51E-03	2.539	2.826	0.057	0.415
**KCNMB2**	c79643_g1	17.139	4.099	4.14E-10	1.80E-05	0.483	0.042	5.114	6.699
**FAR2**	c2722_g1	20.265	4.341	1.15E-06	4.56E-03	0.051	0.032	1.204	0.676

id—contig name; FC—fold change; log2FC—normalized fold change; pval—p value; padj—adjusted p value; Cf_s1, Cf_s2, Cf_a1, Cf_a2—TMM normalized FPKM values in each sample.

The best-saturated mammalian functional annotations (human reference) were used to associate DEGs with 2041 GO terms and 28 DEGs with 41 KEGG (http://www.genome.jp/kegg-bin/) and 208 Reactome (http://www.reactome.org/cgi-bin) metabolic pathway processes (S5 Table and S6 Table).

### Comparison of RNA-Seq and qRT-PCR values

**Fig 5:**
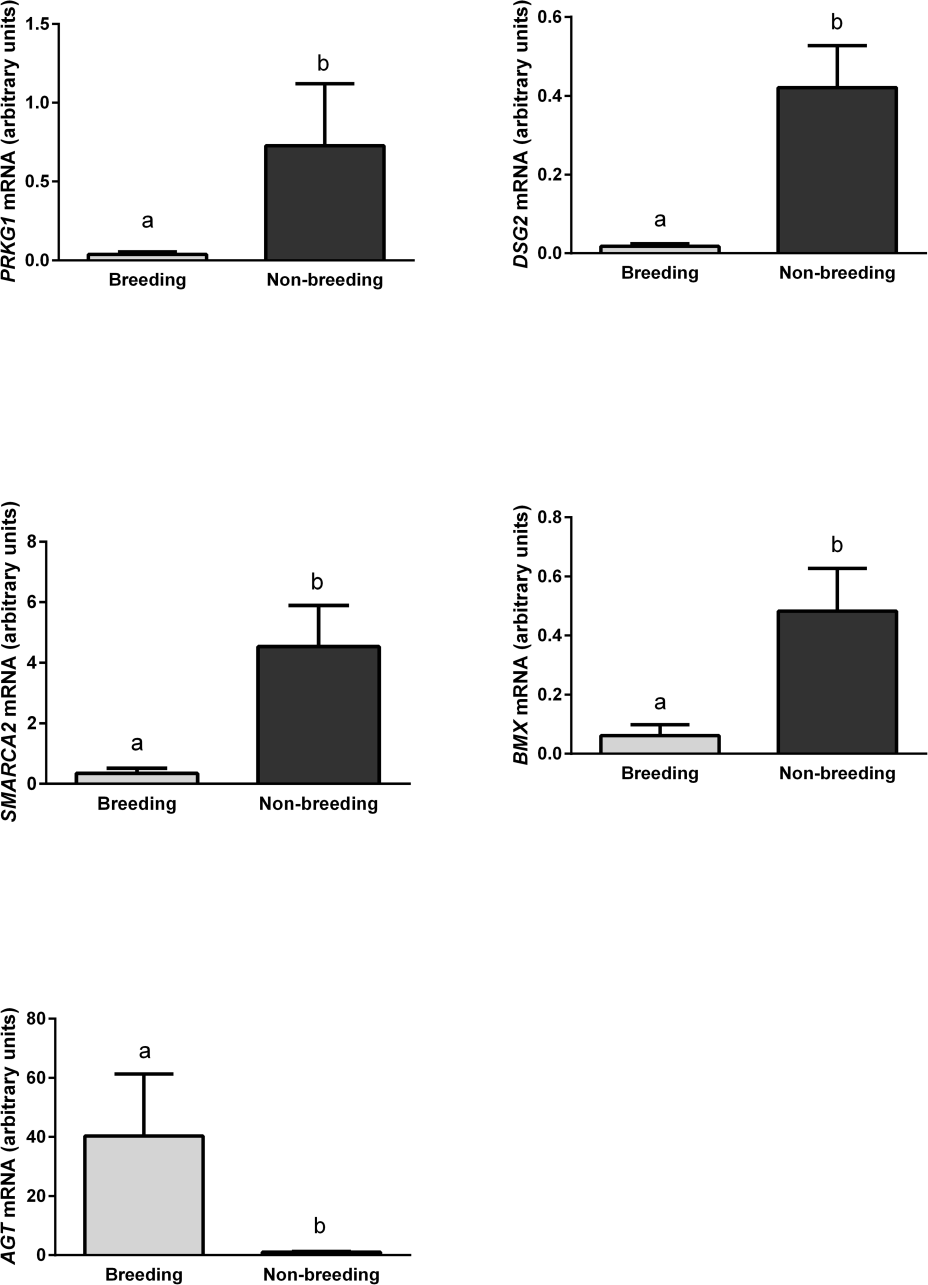
Validation of a quantitative real-time PCR assay. The expression of *PRKG1*, *DSG2*, *SMARCA2* and *BMX* genes was up-regulated, whereas AGT gene expression was down-regulated in beaver testes during the non-breeding season relative to the breeding season. Different letters indicate statistically significant differences (p < 0.05) between the tested groups of beavers.

An analysis of mRNA abundance in genes evaluated by qRT-PCR revealed a correlation with RNA-Seq results (Fig 5). The mRNA levels *PRKG1*, *DSG2*, *SMARCA2 and BMX* were higher and *AGT* expression was lower in November than in April.

## Discussion

To the best of our knowledge, this is the first study providing a comprehensive analysis of transcriptome profiles of European beaver testes with the use of the RNA-Seq technique. It is also the first study to describe variations in transcriptome profiles between breeding and non-breading seasons. The RNA-Seq technique is a powerful tool for generating large-scale transcriptome data that were useful for presenting differentially expressed genes in various species, tissues or cell types. According to some studies, Illumina sequencing data are replicable with relatively little technical variation [35, 36].

In the current study, we obtained a total of 373.06 million high-quality reads (98.12% Q2 bases). *De novo* assembly of contigs yielded 130,741 unigenes which can be useful in future studies of functional genomics in the European beaver. The average length of unigenes with N50 value of 1,734 was 1,369.3, and it was higher than that reported by other researchers [37–40]. The above indicates that our transcriptome sequencing results were successfully assembled and that they are of high quality.

A comprehensive analysis of the testicular transcriptome revealed more than 26,000 highly expressed unigenes which exhibited the highest homology with *Rattus norvegicus* and *Ictidomys tridecemlineatus* genomes and lower homology with other rodent species such as *Dipodomys ordii* or *Mus musculus*. More than 8,000 highly expressed beaver genes were found to be involved in fundamental biological processes, cellular components or molecular pathways. The largest group of genes encoding biological processes comprises genes responsible for the regulation of metabolic processes, transcription and regulation of transcription. A set of genes encoding male reproductive functions was also identified. This group comprises genes controlling the development and proliferation of Sertoli cells, spermatoid differentiation, sperm development, sperm motility and the steroid hormone mediated signaling pathway. Some testis-specific markers involved in beaver reproductive functions could be omitted if they did not meet stringent search criteria, i.e. if they did not show at least a two-fold change in the expression level.

The present study revealed 42 genes that were differentially regulated in the testes during the breeding season than in the non-breeding season. Surprisingly, during the non-breeding season (November), a much larger group of genes (37) was significantly more highly expressed, and a smaller group of genes (5) was less expressed than during the breeding season (April). The up-regulation of a larger number of genes in mouse testes after androgen withdrawal was determined in a microarray analysis by Petrusz *et al*. [41] and Sadate-Ngatchou *et al*. [42]. In the present study, the observed variations in the transcript profiles of beaver testes could be linked with plasma androgens concentrations. However, in our previous study of beavers, plasma testosterone levels did not differ significantly between the analyzed reproductive seasons [16].

In the group of differentially regulated high-impact genes, 18 genes encoded signaling molecules, 7 genes encoded transcription factors and DNA repair molecules, 9 genes encoded cell surface proteins, 10 genes encoded stress response or inflammatory process, 12 genes encoded ion channels and 4 genes encoded extracellular matrix components. Only the most interesting genes are briefly discussed below.

Several up-regulated transcripts from the non-breading season could be involved in the regulation of spermatogenesis in beaver testes, including *VLDLR* which belongs to the low-density lipoprotein receptor (LDLR) family. It has been reported that *VLDLR* overexpression in germ cells disrupted spermatogenesis in transgenic mice [43], which suggests that the *VLDLR* transgene inhibits the development of sperm cells. Similar conclusions could be drawn from our results which showed markedly higher transcript levels in beaver testes during the non-breeding season when spermatogenesis is limited.

*SMAD* proteins are intracellular mediators of the transforming growth factor-β (TGFβ). These transcription factors are implicated in bone morphogenetic protein (BMP) and activin signaling, they are highly involved in the regulation of male fertility, and they influence germ and somatic cells during fetal and postnatal life [44, 45]. In 15-day-old mice, the *SMAD5* transcript was identified by *in situ* hybridization in spermatogonia, whereas a weak signal was detected in spermatocytes. In adult testes, the signal was strongest in spermatogonia and spermatocytes and weaker in round spermatids and in Sertoli cells [46]. Interestingly, our findings revealed up-regulation of the *SMAD5* transcript in beaver testes during the non-breeding season, which could indicate that the *BMPs/SMADs* pathway could play a role in seasonally dependent reproductive activity of the beaver.

The present findings indicate seasonally dependent changes in the expression of genes encoding transporter proteins. The expression of *SLC7A1* and *KCNMB2* (BK channels) in beaver testes was significantly higher in November. The *SLC7A1* gene mediates the transport of cationic amino acids across cell membrane. It has been reported that rat Sertoli cells express *SLC7A1* which relies on the NA+-independent transport system to deliver arginine or other cationic amino acids to germ cells [47]. The *KCNMB2* gene is a calcium-gated potassium channel that has been described in various mammalian endocrine cells, including human and hamster testicular Leydig cells [48]. It has been suggested that these channels contribute to Leydig cell steroidogenesis. Interestingly, the presence of iberiotoxin, a specific channel blocker, did not induce changes in testosterone production by hamster Leydig cells *in vitro* under basal conditions, but a significant increase in testosterone levels was reported when hCG was added to culture media [48]. According to Gong *et al*. [49], these ion channels could also play an important role in spermatogenesis control. Sperm cells could possess a Ca2+-activated K+ channel which has been implicated in sperm activation and gamete interaction [50]. The above findings indicate that changes in the membrane potential of germ cells could be an important element of the signaling mechanism.

The large heparan sulfate proteoglycan (HSPG2, perlecan) is an extracellular matrix component that is normally expressed in the basement membrane (BM) underlying epithelial and endothelial cells. It was detected in the seminiferous tubule BM and in the interstitium, where the protein was localized around blood vessels and Leydig cells [51]. HSPG2 could also play an important role in self-renewal and differentiation of spermatogonial stem cells in the testes [51, 52].

Receptor tyrosine kinase *ErbB4* has been detected on circulating human monocytes and neuronal macrophages, which points to its involvement in inflammatory processes. *ErbB4* was also highly expressed in mouse Sertoli cells and in germ and Leydig cells [53]. Interestingly, targeted inactivation of *ErbB4* in Sertoli cells induced a reduction in testis size, decreased testicular androgen production and delayed spermatogenesis [53].

The down-regulated transcripts identified in our study encode proteins that could be involved in stress responses and inflammatory processes. *IRAK1BP1* is an inhibitory component of TLR, IL-1 and TNFR-related pathways [54, 55]. Overexpression of IRKA1BP1 in macrophages increased the expression of anti-inflammatory IL-10, but decreased the synthesis of proinflammatory IL-6. *IRKA1BP1* also contributes to LPS-induced tolerance by influencing NF-ĸB [55].

In this study, the identified down-regulated genes also included angiotensinogen gene (*AGT*) which is a part of the rennin-angiotensin system. In rat testes, only renin mRNA was detected, whereas both renin and angiotensinogen mRNA were found in mouse testes [56]. The AGT transcript was identified as one of the up-regulated genes (2.64-fold increase, microarray analysis) in the testes of transgenic mice carrying the androgen-binding protein (ABP) gene [41]. The importance of the angiotensin-converting enzyme (ACE, a component of the rennin-angiotensin system) for sperm functions has been demonstrated in male mice by targeting genes that lack somatic ACE but retain testicular ACE [57]. Interestingly, we found that the *LNPEP* (*IRAP*) gene, which is involved in the rennin-angiotensin system, was up-regulated in beaver testes outside the breeding season. The *LNPEP* gene encodes zinc-dependent aminopeptidase, cleaves vasopressin, oxytocin and bradykinin, and catalyzes the final step in the conversion of angiotensinogen to angiotensin IV. The results of our study indicate that angiotensin could be produced locally in beaver testes, and they provide important insights into the biology of the renin-angiotensin system in this species.

In summary, this is the first study to generate a representative testicular transcriptome of the European beaver with the use of the powerful RNA-Seq technique. It is also the first study to describe seasonally dependent variations in the transcriptome profiles of beaver testes. We identified a set of 42 genes encoding molecules that are involved in signal transduction, DNA repair, stress responses, inflammatory processes, metabolism and steroidogenesis. Our findings pave the way for further research into the processes that occur in beaver testes during various periods of reproductive activity.

## Supporting information

S1 Fig KEGG analysis for highly expressed unigenes. (TIF)

S1 Table Characteristics of primers used for real time PCR to validate RNA-Seq transcriptome sequencing. (DOCX)

S2 Table Highly expressed genes (FPKM >2) involved in the biological processes. Gene Ontology terms related to spermatogenesis, steroid hormone mediated signaling pathway, sperm motility, male gonad development, Sertoli cell development, spermatid development, spermatid differentiation, sperm axoneme assembly and Sertoli cell proliferation using the Blastx-fast algorithm in the Blast2GO tool. The columns contain the following information: “SeqName”–the ID of contigs, “Description”–full protein names, “Length”–contig length values, “sim mean”–average similarity between the aligned sequences.
(DOCX)

S3 Table Highly expressed unigenes (FPKM >2) assigned to 116 KEGG pathways. (DOCX)

S4 Table Highly expressed genes (FPKM >2) associated with the steroid hormone biosynthesis KEGG pathway (Blastx-fast algorithm in the Blast2GO tool). The columns contain the following information: “SeqName”–the ID of contigs, “Description”–full protein names, “Length”–contig length values, “sim mean”–average similarity between the aligned sequences.
(DOCX)

S5 Table Functional annotation of differentially expressed genes based on Blast2GO results. (DOCX)

S6 Table Functional annotation of differentially expressed genes based on GO, KEGG and Reactome human databases. The columns contain the following information: “Term”–names of terms in each database, “Database”–database name, “ID”–the id of terms in each database, “Input number”–number of assigned unigenes, “Background number”–number of all genes assigned to a given term in each database, “p-Value”,”Corrected p-Value”–statistical significance of enrichment, “Input”–short names of assigned DEGs.
(XLS)
