# Supplementary figures and images for: Seasonal differences in the testicular transcriptome profile of free-living European beavers (Castor fiber L.) determined by the RNA-Seq method
Source: PLoS One. 2017 Jul 5;12(7):e0180323. doi: 10.1371/journal.pone.0180323 (PMC5498055; doi:10.1371/journal.pone.0180323)

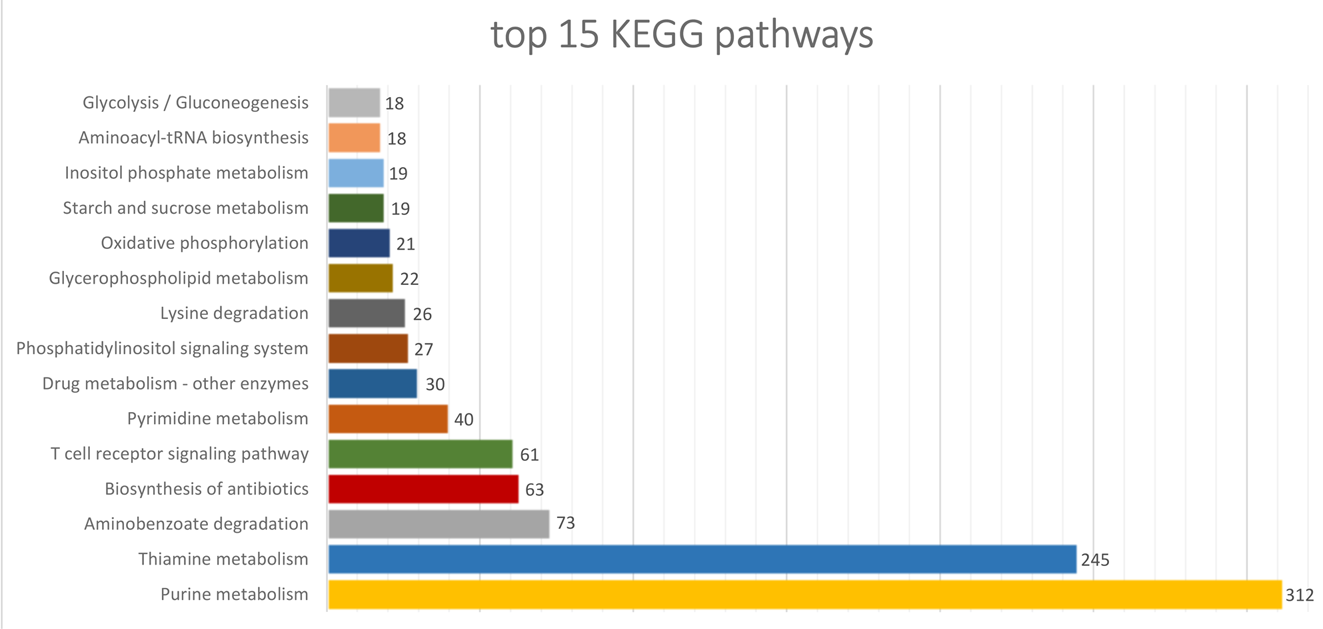

Supplement: S1 Fig — (TIF) [file pone.0180323.s001.tif]
