# Supplementary material for: Seasonal differences in the testicular transcriptome profile of free-living European beavers (Castor fiber L.) determined by the RNA-Seq method
Source: PLoS One. 2017 Jul 5;12(7):e0180323. doi: 10.1371/journal.pone.0180323 (PMC5498055; doi:10.1371/journal.pone.0180323)
Supplement: S1 Table — (DOCX) [file pone.0180323.s002.docx]

S1 Table. **Characteristics of primers used for real time PCR to validate RNA-Seq transcriptome sequencing.**

| Real-Time PCR |  | |  |  |
| --- | --- | --- | --- | --- |
| Gene  name | Primer sequences (5’-3’) | | Tm  (^o^C) | Product  length (bp) |
| *SMARCA2* | F: CTGAAGAGAATGCCGAGGGA  R: GGAGCAACTTCATAACCAGGATTC | | 60 | 196 |
| *DSG2* | F: CCGGCGAAATTTATACAACCAGTA  R: TTTCTTCAACCGTCCCTTCATACA | | 60 | 201 |
| *BMX* | F: TTCAAATACAGCAGCAAGTCAGATG  R: GGAAGATAAGAGTTGCTGGAATGTG | | 60 | 237 |
| *AGT* | F: TCATCACGACCTCCTGACCTG  R: TTCAAAGAGAACGCTATTCAGTACCTC | | 60 | 211 |
| *PRKG1* | F: GTCACTGGTGTATGTCATGGAAGATG  R: TGTCGATCAATAGCCCAGAGTTTC | | 60 | 174 |
| *ACTB* | | F: ATCGCCGACAGGATGCA  R: CGTACTCCTGCTTGCTGATCC | 60 | 102 |
|  |  | |  |  |
| *GAPDH* | F: CCTTCATTGACCTCCACTAC  R: CCACAACATACGTAGCACCA | | 59 | 123 |
