# Supplementary material for: Seasonal differences in the testicular transcriptome profile of free-living European beavers (Castor fiber L.) determined by the RNA-Seq method
Source: PLoS One. 2017 Jul 5;12(7):e0180323. doi: 10.1371/journal.pone.0180323 (PMC5498055; doi:10.1371/journal.pone.0180323)
Supplement: S2 Table — Gene Ontology terms related to spermatogenesis, steroid hormone mediated signaling pathway, sperm motility, male gonad development, Sertoli cell development, spermatid development, spermatid differentiation, sperm axoneme assembly and Sertoli cell proliferation using the Blastx-fast algorithm in the Blast2GO tool. The columns contain the following information: “SeqName”–the ID of contigs, “Description”–full protein names, “Length”–contig length values, “sim mean”–average similarity between the aligned sequences. (DOCX) [file pone.0180323.s003.docx]

S2 Table. **Highly expressed genes (FPKM >2) involved in the biological processes.** Gene Ontology terms related to spermatogenesis, steroid hormone mediated signaling pathway, sperm motility, male gonad development, Sertoli cell development, spermatid development, spermatid differentiation, sperm axoneme assembly and Sertoli cell proliferation using the Blastx-fast algorithm in the Blast2GO tool. The columns contain the following information: “SeqName” – the ID of contigs, “Description” – full protein names, “Length” – contig length values, “sim mean” – average similarity between the aligned sequences.

| **SPERMATOGENESIS** | | | | |
| --- | --- | --- | --- | --- |
| **SeqName** | **Description** | **Length** | **e-Value** | **sim mean** |
| c3960_g1_i1 | angiotensin converting partial | 790 | 8.01E-35 | 95% |
| c7533_g1_i1 | B-cell receptor-associated 31 [Ictidomys tridecemlineatus] | 1305 | 1.67E-117 | 98% |
| c17875_g1_i1 | rac GTPase-activating 1 | 3505 | 0,00E+00 | 98% |
| c27953_g1_i1 | CUGBP Elav-like family member 3 isoform X13 | 1495 | 2.27E-55 | 100% |
| c40649_g1_i1 | spermatogenesis-associated 16 | 2099 | 0,00E+00 | 96% |
| c48069_g1_i2 | cation channel sperm-associated 1 | 2557 | 0,00E+00 | 87% |
| c59227_g1_i1 | intraflagellar transport 81 homolog | 2842 | 0,00E+00 | 99% |
| c61292_g1_i4 | sex comb on midleg homolog isoform partial | 3622 | 0,00E+00 | 96% |
| c62582_g2_i2 | peroxiredoxin-4 isoform X1 | 1162 | 1.25E-160 | 93% |
| c62763_g1_i2 | transcription factor-like 5 isoform X1 | 1003 | 3.61E-10 | 79% |
| c66881_g1_i1 | large proline-rich BAG6 isoform X4 | 3787 | 0,00E+00 | 96% |
| c70554_g1_i1 | PREDICTED: protein DPCD | 1597 | 2.56E-127 | 88% |
| c71109_g1_i1 | testis-specific serine threonine- kinase 3 [Rattus norvegicus] | 2706 | 0,00E+00 | 99% |
| c71848_g2_i1 | centrin-2 isoform X1 [Rattus norvegicus] | 635 | 5.12E-85 | 80% |
| c72945_g1_i2 | plasma serine protease inhibitor [Ictidomys tridecemlineatus] | 3529 | 0,00E+00 | 86% |
| c73051_g1_i1 | ATP-dependent RNA helicase TDRD9 | 4992 | 0,00E+00 | 94% |
| c73536_g1_i5 | polyadenylate-binding -interacting isoform partial | 2213 | 5.66E-51 | 98% |
| c79740_g3_i1 | proteasome activator complex subunit 4 | 7213 | 0,00E+00 | 99% |
| c81043_g1_i5 | trinucleotide repeat containing isoform partial | 1742 | 0,00E+00 | 91% |
| c81874_g2_i1 | doublesex- and mab-3-related transcription factor 1 | 2549 | 1.52E-157 | 91% |
| c82051_g2_i3 | dnaJ homolog subfamily A member 1 [Ictidomys tridecemlineatus] | 3686 | 0,00E+00 | 100% |
| c82712_g1_i1 | 60S ribosomal L10-like [Rattus norvegicus] | 1751 | 1.36E-150 | 100% |
| c83435_g1_i2 | WD repeat-containing 48 isoform X1 | 4167 | 0,00E+00 | 97% |
| c83891_g4_i1 | activin receptor type-2A [Cricetulus griseus] | 2607 | 0,00E+00 | 100% |
| c83961_g6_i2 | bromodomain testis-specific | 4389 | 0,00E+00 | 88% |
| c84368_g2_i1 | Wilms tumor homolog | 1197 | 1.86E-77 | 85% |
| c84368_g3_i1 | truncated Wilms tumor | 1400 | 3.49E-169 | 98% |
| c84651_g2_i2 | Bcl2-like isoform partial | 3215 | 1.53E-164 | 97% |
| c85213_g1_i2 | DNA mismatch repair Mlh1 isoform X1 | 3904 | 0,00E+00 | 94% |
| c86009_g1_i4 | transcription factor SOX-30 isoform X1 | 4079 | 0,00E+00 | 90% |
| c86088_g1_i8 | HORMA domain-containing 1 | 4000 | 8.19E-174 | 96% |
| c86738_g3_i3 | circadian locomoter output cycles kaput isoform X1 [Ictidomys tridecemlineatus] | 2659 | 0,00E+00 | 98% |
| c87326_g1_i1 | AT-rich interactive domain-containing 4B isoform X2 | 5482 | 0,00E+00 | 96% |
| c87410_g5_i3 | synaptonemal complex 3 | 1863 | 8.29E-121 | 91% |
| c89724_g1_i7 | phosphoacetylglucosamine mutase isoform X1 | 5357 | 0,00E+00 | 92% |
| c89970_g1_i1 | tudor domain-containing 1 isoform X1 | 5005 | 0,00E+00 | 92% |
| c90004_g7_i9 | ATP-dependent RNA helicase TDRD12 [Rattus norvegicus] | 5958 | 0,00E+00 | 87% |
| c90324_g12_i13 | KASH5 isoform X1 | 2560 | 3.75E-124 | 93% |
| c90494_g5_i9 | PREDICTED: protein CIP2A | 4226 | 0,00E+00 | 97% |
| c90965_g1_i2 | AT-rich interactive domain-containing 4A isoform X2 | 5212 | 0,00E+00 | 95% |
| c91254_g13_i2 | polyhomeotic 2 isoform X2 | 5930 | 0,00E+00 | 94% |
| c91649_g9_i1 | follicle-stimulating hormone receptor isoform X1 | 4461 | 0,00E+00 | 96% |
| c91729_g1_i3 | cyclin-dependent kinase 16 isoform X4 | 3191 | 0,00E+00 | 96% |
| c91932_g1_i2 | KPL2 isoform CRA_b | 1269 | 3.75E-158 | 88% |
| c92556_g4_i5 | nucleoporin NDC1 | 6076 | 0,00E+00 | 94% |
| c92787_g6_i3 | boule-like (Drosophila) isoform partial | 4908 | 0,00E+00 | 94% |
| c93020_g2_i9 | bone morphogenetic 8a | 2451 | 6.74E-30 | 92% |
| c93179_g3_i8 | E3 ubiquitin- ligase UBR2 isoform X2 | 7884 | 0,00E+00 | 97% |
| c93292_g12_i1 | sphingosine-1-phosphate lyase 1 | 4159 | 0,00E+00 | 96% |
| c93341_g4_i4 | male-enhanced antigen 1 isoform X1 [Ictidomys tridecemlineatus] | 1388 | 1.03E-82 | 96% |
| c93341_g5_i2 | male-enhanced antigen 1 isoform X2 | 768 | 4.15E-86 | 99% |
| c93427_g4_i1 | Fanconi anemia group F | 2927 | 1.4E-147 | 83% |
| c93703_g2_i3 | heat shock-related 70 kDa 2 | 3388 | 0,00E+00 | 99% |
| c93920_g5_i2 | E3 ubiquitin- ligase HERC2 | 15389 | 0,00E+00 | 98% |
| c95417_g5_i1 | breast cancer type 2 susceptibility | 3565 | 0,00E+00 | 87% |
| c95670_g1_i3 | pumilio homolog 1 isoform X12 [Mus musculus] | 8212 | 1.14E-23 | 71% |
|  |  |  |  |  |
| **STEROID HORMONE MEDIATED SIGNALING PATHWAY** | | | | |
| **SeqName** | **Description** | **Length** | **e-Value** | **sim mean** |
| c67283_g1_i1 | retinoic acid receptor RXR-alpha | 5434 | 0,00E+00 | 100% |
| c75618_g1_i3 | steroid hormone receptor ERR2 isoform X1 | 1749 | 0,00E+00 | 100% |
| c79953_g2_i3 | thyroid hormone receptor alpha [Cavia porcellus] | 3155 | 0,00E+00 | 100% |
| c81055_g1_i4 | estrogen receptor partial | 1074 | 3.34E-23 | 97% |
| c83051_g1_i2 | COUP transcription factor 2 isoform X2 [Cavia porcellus] | 2467 | 0,00E+00 | 100% |
| c86261_g1_i5 | Steroid hormone receptor ERR1 | 4014 | 1.41E-128 | 100% |
| c86375_g2_i1 | nuclear receptor subfamily 2 group C member 1 [Ictidomys tridecemlineatus] | 3308 | 0,00E+00 | 97% |
| c86803_g2_i12 | bile acid receptor isoform 1 | 2271 | 2.03E-17 | 95% |
| c87066_g8_i2 | isoform CRA_c | 5505 | 0,00E+00 | 96% |
| c87066_g9_i1 | peroxisome proliferator-activated receptor delta | 2424 | 0,00E+00 | 100% |
| c87472_g2_i3 | retinoic acid receptor-alpha | 4287 | 0,00E+00 | 99% |
| c89555_g3_i11 | retinoic acid receptor RXR-beta isoform 3 [Mus musculus] | 3624 | 2.97E-129 | 97% |
| c90973_g7_i5 | peroxisome proliferator activated receptor isoform partial | 2825 | 0,00E+00 | 94% |
| c93332_g4_i2 | Nuclear receptor subfamily group member 1 | 2794 | 0,00E+00 | 95% |
| c93929_g4_i6 | Estrogen receptor | 5563 | 1.13E-16 | 100% |
| c94820_g4_i6 | nuclear receptor subfamily 1 group I member 2 | 3566 | 2.62E-146 | 88% |
| c95287_g2_i1 | oxysterols receptor LXR-beta isoform X3 [Heterocephalus glaber] | 2157 | 0,00E+00 | 92% |
|  |  |  |  |  |
| **SPERM MOTILITY** | | | | |
| **SeqName** | **Description** | **Length** | **e-Value** | **sim mean** |
| c27953_g1_i1 | CUGBP Elav-like family member 3 isoform X13 | 1495 | 2.27E-55 | 100% |
| c43536_g1_i1 | SRA stem-loop-interacting RNA-binding mitochondrial | 1463 | 1.25E-56 | 88% |
| c48069_g1_i2 | cation channel sperm-associated 1 | 2557 | 0,00E+00 | 87% |
| c62052_g1_i3 | inhibitor of growth 2 | 1289 | 7.93E-99 | 98% |
| c70554_g1_i1 | PREDICTED: protein DPCD | 1597 | 2.56E-127 | 88% |
| c75541_g1_i1 | probable ATP-dependent RNA helicase DDX4 isoform X4 | 698 | 4.99E-122 | 87% |
| c81043_g1_i5 | trinucleotide repeat containing isoform partial | 1742 | 0,00E+00 | 91% |
| c82051_g2_i3 | dnaJ homolog subfamily A member 1 [Ictidomys tridecemlineatus] | 3686 | 0,00E+00 | 100% |
| c84368_g2_i1 | Wilms tumor homolog | 1197 | 1.86E-77 | 85% |
| c84368_g3_i1 | truncated Wilms tumor | 1400 | 3.49E-169 | 98% |
| c87070_g1_i3 | tubulin polyglutamylase TTLL5 isoform X1 | 5028 | 0,00E+00 | 96% |
| c89435_g1_i1 | probable ATP-dependent RNA helicase DDX4 isoform X1 | 4466 | 0,00E+00 | 96% |
| c89944_g9_i9 | phospholipid transfer | 3162 | 0,00E+00 | 90% |
| c92690_g1_i1 | mKIAA0673 protein | 5520 | 0,00E+00 | 93% |
| c95510_g6_i13 | dynein heavy chain axonemal | 2218 | 1.7E-48 | 100% |
|  |  |  |  |  |
| **MALE GONAD DEVELOPMENT** | | | | |
| **SeqName** | **Description** | **Length** | **e-Value** | **sim mean** |
| c14645_g2_i1 | histone [Heterocephalus glaber] | 1640 | 2.01E-87 | 100% |
| c35119_g1_i1 | DNA mismatch repair Msh2 | 3258 | 0,00E+00 | 97% |
| c50064_g1_i1 | testis-expressed sequence 11 | 949 | 3.25E-31 | 90% |
| c50362_g1_i1 | LIM homeobox Lhx9 isoform X3 | 1317 | 2.57E-28 | 98% |
| c62582_g2_i2 | peroxiredoxin-4 isoform X1 | 1162 | 1.25E-160 | 93% |
| c67626_g1_i3 | kit ligand isoform X2 | 5324 | 2.19E-144 | 93% |
| c76754_g1_i1 | Tescalcin ( ) isoform CRA_a | 1264 | 4.85E-63 | 99% |
| c83109_g1_i3 | AT-rich interactive domain-containing 5B isoform X1 | 7557 | 0,00E+00 | 96% |
| c84651_g2_i2 | Bcl2-like isoform partial | 3215 | 1.53E-164 | 97% |
| c84679_g3_i1 | transcription factor GATA-6 | 1995 | 2.65E-19 | 97% |
| c87066_g8_i2 | isoform CRA_c | 5505 | 0,00E+00 | 96% |
| c89467_g1_i8 | vascular endothelial growth factor receptor 2 isoform X1 | 1196 | 8.75E-13 | 94% |
| c92394_g10_i2 | inhibin beta A chain [Mesocricetus auratus] | 2076 | 0,00E+00 | 98% |
|  |  |  |  |  |
| **SERTOLI CELL DEVELOPMENT** | | | | |
| **SeqName** | **Description** | **Length** | **e-Value** | **sim mean** |
| c81874_g2_i1 | doublesex- and mab-3-related transcription factor 1 | 2549 | 1.52E-157 | 91% |
| c84368_g2_i1 | Wilms tumor homolog | 1197 | 1.86E-77 | 85% |
| c84368_g3_i1 | truncated Wilms tumor | 1400 | 3.49E-169 | 98% |
| c87463_g4_i3 | fibronectin type-III domain-containing 3A isoform X1 | 4255 | 0,00E+00 | 97% |
| c91649_g9_i1 | follicle-stimulating hormone receptor isoform X1 | 4461 | 0,00E+00 | 96% |
|  |  |  |  |  |
| **SPERMATID DEVELOPMENT** | | | | |
| **SeqName** | **Description** | **Length** | **e-Value** | **sim mean** |
| c14645_g2_i1 | histone [Heterocephalus glaber] | 1640 | 2.01E-87 | 100% |
| c30833_g1_i1 | ATP-dependent RNA helicase DDX25 [Mus musculus] | 1794 | 0,00E+00 | 99% |
| c43536_g1_i1 | SRA stem-loop-interacting RNA-binding mitochondrial | 1463 | 1.25E-56 | 88% |
| c50111_g1_i1 | parkin coregulated gene | 1620 | 4.99E-177 | 100% |
| c60898_g1_i1 | calcium and integrin-binding 1 [Rattus norvegicus] | 1428 | 1.26E-87 | 88% |
| c61998_g1_i2 | zinc finger MYND domain-containing 15 isoform X2 | 4065 | 0,00E+00 | 94% |
| c62052_g1_i3 | inhibitor of growth 2 | 1289 | 7.93E-99 | 98% |
| c70698_g1_i1 | homeobox SIX5 | 2738 | 0,00E+00 | 91% |
| c73637_g1_i1 | AF467890_1 quaking II | 2382 | 4.79E-8 | 74% |
| c76396_g1_i4 | Hook homolog 1 isoform X1 | 2833 | 0,00E+00 | 97% |
| c77726_g2_i1 | oxysterol-binding 2 [Rattus norvegicus] | 3887 | 0,00E+00 | 84% |
| c78923_g1_i2 | testis-specific serine threonine- kinase 2 | 3676 | 0,00E+00 | 96% |
| c82349_g1_i1 | PREDICTED: P protein | 3207 | 0,00E+00 | 90% |
| c83337_g1_i7 | nucleoside diphosphate kinase homolog 5 | 1263 | 4.11E-141 | 97% |
| c86455_g2_i4 | Alstrom syndrome 1 | 5235 | 0,00E+00 | 83% |
| c86526_g1_i2 | qkI-7 [Mus musculus] | 6425 | 0,00E+00 | 100% |
| c87463_g4_i3 | fibronectin type-III domain-containing 3A isoform X1 | 4255 | 0,00E+00 | 97% |
| c87970_g4_i4 | Bardet-Biedl syndrome 4 isoform X1 | 3007 | 0,00E+00 | 96% |
| c88904_g6_i2 | CUG triplet RNA binding isoform CRA_c | 3730 | 8.38E-11 | 100% |
| c90245_g6_i2 | tRNA (cytosine(34)-C(5))-methyltransferase | 2819 | 0,00E+00 | 93% |
| c91647_g11_i1 | truncated tyrosine kinase receptor [Mus musculus] | 674 | 4.27E-98 | 97% |
| c91870_g3_i6 | spermatid perinuclear RNA-binding | 4160 | 0,00E+00 | 99% |
| c92191_g11_i7 | CUG triplet RNA binding isoform partial [Mus musculus] | 4002 | 0,00E+00 | 98% |
| c92659_g5_i5 | disrupted meiotic cDNA 1 isoform partial | 1732 | 3.33E-86 | 95% |
| c94059_g2_i6 | IQ domain-containing G | 2580 | 0,00E+00 | 87% |
| c94812_g3_i4 | usick-Kaufman Bardet-Biedl syndromes chaperonin | 4042 | 0,00E+00 | 92% |
| c95586_g4_i3 | meiosis inhibitor 1 | 2460 | 0,00E+00 | 89% |
|  |  |  |  |  |
| **SPERMATID DIFFERENTIATION** | | | | |
| **SeqName** | **Description** | **Length** | **e-Value** | **sim mean** |
| c675_g1_i1 | transmembrane 119 | 2214 | 1.13E-85 | 85% |
| c66445_g1_i4 | Bcl2-associated X isoform partial | 1328 | 1.38E-115 | 97% |
|  |  |  |  |  |
| **SPERM AXONEME ASSEMBLY** | | | | |
| **SeqName** | **Description** | **Length** | **e-Value** | **sim mean** |
| c85153_g1_i1 | Bardet-Biedl syndrome 2 | 2719 | 0,00E+00 | 96% |
| c87070_g1_i3 | tubulin polyglutamylase TTLL5 isoform X1 | 5028 | 0,00E+00 | 96% |
| c92889_g11_i7 | sperm-associated antigen 16 | 2282 | 0,00E+00 | 92% |
|  |  |  |  |  |
| **SERTOLI CELL PROLIFERATION** | | | | |
| **SeqName** | **Description** | **Length** | **e-Value** | **sim mean** |
| c66445_g1_i4 | Bcl2-associated X isoform partial | 1328 | 1.38E-115 | 97% |
| c83891_g4_i1 | activin receptor type-2A [Cricetulus griseus] | 2607 | 0,00E+00 | 100% |
| c91649_g9_i1 | follicle-stimulating hormone receptor isoform X1 | 4461 | 0,00E+00 | 96% |
|  |  |  |  |  |
