# Supplementary material for: Seasonal differences in the testicular transcriptome profile of free-living European beavers (Castor fiber L.) determined by the RNA-Seq method
Source: PLoS One. 2017 Jul 5;12(7):e0180323. doi: 10.1371/journal.pone.0180323 (PMC5498055; doi:10.1371/journal.pone.0180323)
Supplement: S3 Table — (DOCX) [file pone.0180323.s004.docx]

S3 Table. **Highly expressed unigenes (FPKM >2) assigned to 116 KEGG pathways**.

| **Pathway** | **Assigned unigenes** | **Pathway ID** |
| --- | --- | --- |
| Purine metabolism | 312 | map00230 |
| Thiamine metabolism | 245 | map00730 |
| Aminobenzoate degradation | 73 | map00627 |
| Biosynthesis of antibiotics | 63 | map01130 |
| T cell receptor signaling pathway | 61 | map04660 |
| Pyrimidine metabolism | 40 | map00240 |
| Drug metabolism - other enzymes | 30 | map00983 |
| Phosphatidylinositol signaling system | 27 | map04070 |
| Lysine degradation | 26 | map00310 |
| Glycerophospholipid metabolism | 22 | map00564 |
| Oxidative phosphorylation | 21 | map00190 |
| Starch and sucrose metabolism | 19 | map00500 |
| Inositol phosphate metabolism | 19 | map00562 |
| Aminoacyl-tRNA biosynthesis | 18 | map00970 |
| Glycolysis / Gluconeogenesis | 18 | map00010 |
| Pyruvate metabolism | 14 | map00620 |
| Porphyrin and chlorophyll metabolism | 14 | map00860 |
| Sphingolipid metabolism | 14 | map00600 |
| Arachidonic acid metabolism | 13 | map00590 |
| Drug metabolism - cytochrome P450 | 13 | map00982 |
| Metabolism of xenobiotics by cytochrome P450 | 13 | map00980 |
| Arginine and proline metabolism | 12 | map00330 |
| Carbon fixation pathways in prokaryotes | 12 | map00720 |
| Amino sugar and nucleotide sugar metabolism | 12 | map00520 |
| Glutathione metabolism | 12 | map00480 |
| Ether lipid metabolism | 12 | map00565 |
| Alanine, aspartate and glutamate metabolism | 12 | map00250 |
| Steroid hormone biosynthesis | 12 | map00140 |
| Pentose and glucuronate interconversions | 12 | map00040 |
| Cysteine and methionine metabolism | 11 | map00270 |
| Glyoxylate and dicarboxylate metabolism | 11 | map00630 |
| alpha-Linolenic acid metabolism | 10 | map00592 |
| Other glycan degradation | 10 | map00511 |
| Nicotinate and nicotinamide metabolism | 10 | map00760 |
| Ascorbate and aldarate metabolism | 10 | map00053 |
| Fructose and mannose metabolism | 10 | map00051 |
| Retinol metabolism | 10 | map00830 |
| Tryptophan metabolism | 9 | map00380 |
| Arginine biosynthesis | 9 | map00220 |
| Citrate cycle (TCA cycle) | 9 | map00020 |
| Glycerolipid metabolism | 9 | map00561 |
| Phenylalanine metabolism | 9 | map00360 |
| Butanoate metabolism | 9 | map00650 |
| Tyrosine metabolism | 9 | map00350 |
| Propanoate metabolism | 9 | map00640 |
| Pentose phosphate pathway | 8 | map00030 |
| Methane metabolism | 8 | map00680 |
| Carbon fixation in photosynthetic organisms | 8 | map00710 |
| Valine, leucine and isoleucine degradation | 8 | map00280 |
| N-Glycan biosynthesis | 8 | map00510 |
| Fatty acid degradation | 8 | map00071 |
| Linoleic acid metabolism | 7 | map00591 |
| Pantothenate and CoA biosynthesis | 7 | map00770 |
| beta-Alanine metabolism | 7 | map00410 |
| Terpenoid backbone biosynthesis | 7 | map00900 |
| Glycine, serine and threonine metabolism | 7 | map00260 |
| Phenylpropanoid biosynthesis | 7 | map00940 |
| Histidine metabolism | 7 | map00340 |
| mTOR signaling pathway | 7 | map04150 |
| Nitrogen metabolism | 6 | map00910 |
| Other types of O-glycan biosynthesis | 6 | map00514 |
| Various types of N-glycan biosynthesis | 6 | map00513 |
| One carbon pool by folate | 6 | map00670 |
| Galactose metabolism | 6 | map00052 |
| Ubiquinone and other terpenoid-quinone biosynthesis | 5 | map00130 |
| Mucin type O-Glycan biosynthesis | 5 | map00512 |
| Streptomycin biosynthesis | 4 | map00521 |
| Phenylalanine, tyrosine and tryptophan biosynthesis | 4 | map00400 |
| Selenocompound metabolism | 4 | map00450 |
| Biosynthesis of unsaturated fatty acids | 3 | map01040 |
| Chloroalkane and chloroalkene degradation | 3 | map00625 |
| Geraniol degradation | 3 | map00281 |
| Tropane, piperidine and pyridine alkaloid biosynthesis | 3 | map00960 |
| Glycosphingolipid biosynthesis - lacto and neolacto series | 3 | map00601 |
| Synthesis and degradation of ketone bodies | 3 | map00072 |
| Glycosylphosphatidylinositol(GPI)-anchor biosynthesis | 3 | map00563 |
| Novobiocin biosynthesis | 3 | map00401 |
| Cyanoamino acid metabolism | 3 | map00460 |
| Isoquinoline alkaloid biosynthesis | 3 | map00950 |
| Lysine biosynthesis | 3 | map00300 |
| Benzoate degradation | 3 | map00362 |
| Steroid biosynthesis | 3 | map00100 |
| Fatty acid elongation | 3 | map00062 |
| Fatty acid biosynthesis | 3 | map00061 |
| Caprolactam degradation | 3 | map00930 |
| Glycosaminoglycan biosynthesis - keratan sulfate | 3 | map00533 |
| Steroid degradation | 2 | map00984 |
| Lipoic acid metabolism | 2 | map00785 |
| Primary bile acid biosynthesis | 2 | map00120 |
| Limonene and pinene degradation | 2 | map00903 |
| Glycosphingolipid biosynthesis - globo series | 2 | map00603 |
| Folate biosynthesis | 2 | map00790 |
| Glycosaminoglycan biosynthesis - heparan sulfate / heparin | 2 | map00534 |
| Glycosaminoglycan biosynthesis - chondroitin sulfate / dermatan sulfate | 2 | map00532 |
| Glycosaminoglycan degradation | 2 | map00531 |
| Taurine and hypotaurine metabolism | 1 | map00430 |
| Sulfur metabolism | 1 | map00920 |
| Carbapenem biosynthesis | 1 | map00332 |
| Caffeine metabolism | 1 | map00232 |
| Toluene degradation | 1 | map00623 |
| Butirosin and neomycin biosynthesis | 1 | map00524 |
| Polyketide sugar unit biosynthesis | 1 | map00523 |
| Melanogenesis | 1 | map04916 |
| Sesquiterpenoid and triterpenoid biosynthesis | 1 | map00909 |
| Zeatin biosynthesis | 1 | map00908 |
| Indole alkaloid biosynthesis | 1 | map00901 |
| D-Glutamine and D-glutamate metabolism | 1 | map00471 |
| Glycosphingolipid biosynthesis - ganglio series | 1 | map00604 |
| C5-Branched dibasic acid metabolism | 1 | map00660 |
| Cutin, suberine and wax biosynthesis | 1 | map00073 |
| Bisphenol degradation | 1 | map00363 |
| Vitamin B6 metabolism | 1 | map00750 |
| Riboflavin metabolism | 1 | map00740 |
| Styrene degradation | 1 | map00643 |
| Ethylbenzene degradation | 1 | map00642 |
| Biosynthesis of vancomycin group antibiotics | 1 | map01055 |
