# Supplementary material for: Seasonal differences in the testicular transcriptome profile of free-living European beavers (Castor fiber L.) determined by the RNA-Seq method
Source: PLoS One. 2017 Jul 5;12(7):e0180323. doi: 10.1371/journal.pone.0180323 (PMC5498055; doi:10.1371/journal.pone.0180323)
Supplement: S4 Table — The columns contain the following information: “SeqName”–the ID of contigs, “Description”–full protein names, “Length”–contig length values, “sim mean”–average similarity between the aligned sequences. (DOCX) [file pone.0180323.s005.docx]

S4 Table. **Highly expressed genes (FPKM >2) associated with the steroid hormone biosynthesis KEGG pathway** (Blastx-fast algorithm in the Blast2GO tool). The columns contain the following information: “SeqName” - the ID of contigs, “Description” - full protein names, “Length” - contig length values, “sim mean” - average similarity between the aligned sequences.

| **SeqName** | **Description** | **Length** | **e-Value** | **sim mean** |
| --- | --- | --- | --- | --- |
| c7652_g1_i1 | beta-1,4-glucuronyltransferase 1 | 2083 | 0.0E0 | 99% |
| c79848_g2_i1 | PREDICTED: exostosin-1 | 3250 | 0.0E0 | 99% |
| c82644_g2_i2 | PREDICTED: aromatase | 8664 | 1.9E-9 | 91% |
| c82948_g1_i2 | UDP-glucuronosyltransferase 1-8 precursor [Rattus norvegicus] | 2408 | 0.0E0 | 90% |
| c83525_g1_i2 | beta-1,4-galactosyltransferase 5 [Mus musculus] | 3976 | 0.0E0 | 98% |
| c85585_g1_i1 | Dpy19l1 [Mus musculus] | 4199 | 1.3E-117 | 98% |
| c85921_g1_i3 | Sulfotransferase family member 1 [Mus musculus] | 4156 | 5.7E-57 | 98% |
| c88855_g3_i3 | unnamed protein product | 2638 | 1.7E-71 | 92% |
| c90534_g7_i3 | sterol-4-alpha-carboxylate 3- decarboxylating | 2441 | 0.0E0 | 100% |
| c90589_g1_i3 | GnT1IP-L | 2576 | 1.9E-138 | 81% |
| c90826_g2_i5 | arylsulfatase G | 2248 | 0.0E0 | 88% |
| c92717_g2_i10 | glycosyltransferase LARGE1 isoform X2 | 5250 | 0.0E0 | 98% |
