# Supplementary material for: Seasonal differences in the testicular transcriptome profile of free-living European beavers (Castor fiber L.) determined by the RNA-Seq method
Source: PLoS One. 2017 Jul 5;12(7):e0180323. doi: 10.1371/journal.pone.0180323 (PMC5498055; doi:10.1371/journal.pone.0180323)
Supplement: S5 Table — (DOCX) [file pone.0180323.s006.docx]

S5 Table. **Functional annotation of differentially expressed genes** based on Blast2GO results.

| **Function category** | **Functionally assigned unigenes** | **Short names of functionally assigned genes** |
| --- | --- | --- |
| transcript coding enzymes | 9 | USP34; PTPRZ1;ERBB4; PRTN3; TCF20; HCFC1; BMX; DYNC1H1; PRKG1; SMARCA2; UGT8 |
| signaling molecules | 19 | PTPRZ1; ERBB4; FN1; PRKG1; AGT; KCNMB2; VLDLR; SMAD5; BMX; DSG2; ZCCHC11; LNPEP; TNPO1; USP34; RPS27; HSPG2; SNX13; PLEKHG3; IRAK1BP1 |
| DNA repair molecules | 6 | HCFC1; AGT; TCF20; SMAD5; ZCCHC11; SMARCA2 |
| transcription factors | 1 | LKAP |
| surface proteins | 9 | ERBB4; AGT; VLDLR; SMAD5; BMX; DSG2; ZCCHC11; LNPEP; USP34 |
| stress response | 10 | PTPRZ1; ERBB4; FN1; SMAD5; BMX; DYNC1H1; LNPEP; NCR3LG1; PRTN3; IRAK1BP1 |
| inflammatory (immune) process | 2 | KCNMB2; AHNAK |
| ion channels | 10 | ERBB4; FN1; PRKG1; AGT; KCNMB2; VLDLR; BMX; DYNC1H1; PRTN3; LKAP |
| metabolism | 30 | PTPRZ1; UGT8; ERBB4; FN1; PRKG1; HCFC1; AGT; TCF20; VLDLR; SMAD5; BMX; FAR2; ZCCHC11; AHNAK; DYNC1H1; MBNL3; LNPEP; TNPO1; PRTN3; USP34; SMARCA2; RPL17; RPS27; LKAP; HSPG2; SNX13; PLEKHG3; ZBED6; IRAK1BP1; ZNF697 |
| extracellular matrix components | 4 | PTPRZ1; FN1; FREM2; HSPG2 |
